# Strategies for mitigating heat stress and their effects on behavior, physiological indicators, and growth performance in communally managed feedlot cattle

**DOI:** 10.3389/fvets.2025.1513368

**Published:** 2025-02-14

**Authors:** Mhlangabezi Slayi, Ishmael Festus Jaja

**Affiliations:** ^1^Centre for Global Change (CGC), Faculty of Science and Agriculture, University of Fort Hare, Alice, South Africa; ^2^Department of Livestock and Pasture Science, Faculty of Science and Agriculture, University of Fort Hare, Alice, South Africa

**Keywords:** skin temperature, animal welfare, thermal-humidity index, custom feedlots, climate change, fattening performance

## Abstract

Heat stress poses a significant challenge in communal feedlot systems, affecting cattle welfare and productivity. This study evaluated the impact of shade and water-cooling interventions on thermophysiological stress reduction and growth performance in 60 cattle from communal feedlots. Physiological indicators (rectal temperature, skin temperature, respiration rate) along growth metrics (feed intake, average daily gain [ADG]) were analyzed using regression and principal component analysis (PCA) to identify key drivers of performance. The results showed a significant reduction (*p* < 0.05) in rectal temperature, respiration rate, and skin temperature in cattle subjected to shade and water cooling compared to the control group. Temperature-Humidity Index (THI) values frequently exceeded the heat stress threshold of 72, with peak mid-day values surpassing 80, indicating severe thermal stress. Cattle in the treated groups experienced lower THI values, reduced panting scores, and improved homeostasis under high thermal loads. Breed-specific differences were evident, with *Bos indicus* cattle (Nguni) maintaining lower physiological stress indicators than *Bos taurus* (Bonsmara), highlighting superior heat tolerance. Growth performance, measured by average daily gain (ADG) and feed conversion ratio (FCR), significantly improved in the treated groups, with ADG increasing by 18% and FCR improving by 12% relative to the control. Blood metabolite analysis revealed lower cortisol levels (*p* < 0.05) and improved electrolyte balance in the cooled groups, indicating reduced chronic stress and better metabolic function. Behavioral observations, recorded at 10-min intervals every 30 min, showed increased resting time and reduced panting frequency in cooled cattle, confirming enhanced thermal comfort. These findings underscore the importance of integrating cooling interventions into cattle management strategies to improve productivity and welfare in heat-stressed environments.

## 1 Introduction

Communally managed feedlots are essential for improving livestock production and enhancing food security in rural communities, particularly in semi-arid regions characterized by high temperatures, low rainfall, and frequent droughts (1, 2). These regions face significant challenges, including climatic variability and feed scarcity, which often hinder effective livestock management (3). Communal feedlots serve as a cost-effective platform for smallholder farmers, enabling them to collectively manage feed resources, fatten cattle for market, enhance herd health, and generate income, especially during dry seasons when forage availability is critically limited (4, 5).

Despite their benefits, cattle in communal feedlots are exposed to extreme environmental conditions, such as high ambient temperatures and prolonged heat stress, which significantly impair productivity (6). Heat stress is a major concern in semi-arid environments (7), disrupting the normal thermos-physiological processes that help animals maintain homeostasis (8). Prolonged heat exposure compromises the animals’ ability to regulate body temperature, resulting in elevated core body temperatures, increased respiration rates, and higher skin surface temperatures (9, 10). These physiological responses, while aimed at dissipating excess heat, often lead to heat exhaustion, reduced feed intake, and impaired digestive functions such as rumen fermentation (11).

The consequences of heat stress extend beyond reduced growth performance. It weakens immune function, making cattle more susceptible to infections and diseases (12). Prolonged exposure elevates cortisol levels, a stress hormone linked to immunosuppression and heightened vulnerability to pathogens (13). Additionally, heat stress exacerbates water and electrolyte imbalances, further impairing metabolic processes (14). These challenges highlight the urgent need for effective, low-cost heat mitigation strategies, particularly in resource-limited communal feedlot systems where smallholder farmers often lack access to advanced infrastructure (15).

While extensive research has focused on heat stress management in commercial feedlots with advanced cooling technologies (6, 16), little attention has been given to communal feedlots in semi-arid regions. Here, the lack of resources necessitates practical, affordable solutions tailored to the needs of smallholder farmers (5). Promising strategies such as providing shade, implementing water cooling systems, and improving feed supplementation have demonstrated potential in reducing thermal stress and enhancing livestock productivity (16, 17). This study addresses a critical gap by evaluating the thermo-physiological responses, feed intake patterns, and growth performance of communally managed feedlot cattle in semi-arid regions. Specifically, it assesses the effectiveness of adaptive strategies such as shade provision, water cooling, and enhanced feed supplementation in reducing heat stress and promoting cattle health. By focusing on these critical factors, this research aims to develop sustainable livestock management practices that enhance resilience and improve food security for smallholder farmers in semi-arid regions facing the growing threat of climate change.

## 2 Materials and methods

### 2.1 Ethical considerations

This study was conducted in strict accordance with ethical guidelines for animal research and received approval (Reference No: JAJ051SMPO01) from the University of Fort Hare Animal Research Ethics Committee. All procedures involving animals adhered to the institutional ethical standards and were performed with the utmost priority for the welfare of the animals. Measures to minimize stress included providing appropriate housing, nutrition, and environmental enrichment, as well as conducting procedures under the supervision of qualified personnel.

### 2.2 Description of the study area

This study was conducted in communal feedlots located in Centane, Eastern Cape Province, South Africa. The feedlot is situated in a semi-arid region characterized by high temperatures, low annual rainfall, and prolonged dry seasons. The feedlot is positioned at 32.18 degrees south latitude, 28.02 degrees east longitude, with an elevation of 501 meters above sea level. The average annual rainfall in the study area is approximately 450 mm, with peak precipitation occurring between November and March. During the study period, recorded daily temperatures ranged from 28°C to 42°C, with relative humidity fluctuating between 30 and 65%. Peak solar radiation exceeded 900 W/m^2^ around midday. Temperature-Humidity Index (THI) values were continuously monitored using a digital climate station to assess thermal stress levels. To capture diurnal variations in heat stress, microclimatic data (ambient temperature, relative humidity, solar radiation, and wind speed) were recorded at 10-min intervals, every 30 min throughout the study. Data collection occurred from 06:00 to 18:00, with specific attention to critical heat load periods between 12:00 and 15:00. The frequency and duration of high THI values were recorded to determine prolonged exposure to thermal stress conditions. The feedlot is surrounded by villages that grapple with significant socio-economic challenges, including high rates of youth unemployment and dependence on government social grants for support. Subsistence livestock farming and crop cultivation serve as primary sources of income in these resource-constrained communities, playing pivotal roles in sustaining the local population. The communal feedlots are typically managed by local communities, where cattle are brought together for fattening or finishing before being sold (5, 18). The facility has a 2000 maximum livestock carrying capacity. The feedlot management practices include a structured feeding regimen, where cattle are provided with a balanced diet consisting of locally sourced crop residues, silage, and commercial feed supplements to ensure optimal weight gain and fattening. Water is supplied ad libitum through community-managed boreholes and water troughs, ensuring consistent access. Cattle are housed in open-air pens with minimal shelter, exposed to natural elements but with sufficient space for movement. Veterinary care is administered periodically, with routine vaccinations, deworming, and treatment for common diseases to maintain herd health. The environmental conditions in this region make it an ideal site to study the effects of heat stress on cattle. Average daily temperatures range between 25°C and 40°C during the hottest months, with minimal shade and natural cooling options available.

### 2.3 Experimental animals and management

The study involved 60 communal cattle, comprising *Bos taurus* and *Bos indicus* crossbreeds, which were randomly selected from different smallholder farmers based on uniform body weight (350–400 kg) and age (18–24 months). The animals were distributed into three treatment groups of 20 each and managed in a feedlot facility equipped with basic shelter and feed troughs. The control group was maintained without any heat stress mitigation interventions, while the shade group had access to artificial shade structures, and the water-cooling group was subjected to periodic spraying with cool water. All cattle were provided with a balanced diet consisting of locally sourced hay, maize, and commercial concentrates, supplemented with essential minerals and vitamins to meet their nutritional requirements. The study spanned 90 days, during which cattle were monitored for physiological responses, growth performance, and behavioral adaptations.

### 2.4 Data collection

#### 2.4.1 Thermo-physiological measurements

To capture variations in heat stress and thermoregulation, thermos-physiological measurements were taken daily at three time points: early morning (06:00), midday (12:00), and late afternoon (16:00). These intervals were chosen to reflect the effects of diurnal temperature fluctuations on animal physiology. Rectal temperature was measured using a calibrated digital thermometer inserted 8–10 cm into the rectum, providing a reliable indicator of core body temperature. Respiration rate was recorded by visually counting the number of flank movements per minute during a calm observational period, as this parameter reflects evaporative cooling under heat stress. Skin surface temperatures were measured with an infrared thermometer held 10–15 cm from the skin at the neck, back, and flank, capturing peripheral heat dissipation across areas with varying exposure to solar radiation and airflow.

#### 2.4.2 Feed intake and water consumption and growth performance

Feed intake was recorded daily by weighing the feed offered and the refusals at the same time each day, providing an estimate of individual feed consumption. Water intake was monitored by measuring the remaining water in troughs filled to a marked level every morning. These metrics provided insights into the animals’ nutritional intake and hydration, both critical for thermoregulation in hot environments. Growth performance was assessed by weighing the animals at the start of the trial, every two weeks during the trial, and at its conclusion using a digital livestock scale. Average daily gain (ADG) was calculated as the difference between initial and final weights divided by the number of trial days, while feed conversion ratio (FCR) was determined as the ratio of total feed intake to weight gain.

#### 2.4.3 Blood metabolite analysis

Blood samples were collected biweekly from the jugular vein using vacutainer tubes, with sampling performed in the morning before feeding to minimize postprandial variability. Analyses included glucose, urea, and total protein levels to evaluate nutritional and metabolic status, and serum cortisol as a biomarker of chronic physiological stress. Sodium and potassium levels were measured to assess hydration and thermoregulatory efficiency, reflecting electrolyte balance critical for muscle function and osmotic stability. Serum was separated via centrifugation, and metabolite concentrations were determined using automated analyzers and enzyme-linked immunosorbent assay (ELISA) kits.

#### 2.4.4 Behavioral monitoring

Behavioral observations were conducted over an eight-week period to assess welfare and adaptive responses to cooling interventions. Observations were made three times weekly at different times of day, with each session lasting three hours. A combination of focal sampling and video recordings was used, with trained observers monitoring each animal for 10 min at 30-min intervals. Behavioral parameters included resting, feeding, panting, aggressive interactions, and grooming, with panting scored on a scale from 0 (no panting) to 4 (severe panting). Resting and aggressive behaviors were recorded to evaluate comfort and competition for resources, while grooming, such as licking, was observed as a thermoregulatory strategy.

### 2.5 Statistical analysis

Data were analyzed using R software (version 4.0.5) with the tidyverse, stats, FactoMineR, and factoextra packages for statistical analysis and visualization. Thermo-physiological responses, feed intake, growth performance, and blood metabolite levels were compared among treatment groups using analysis of variance (ANOVA), followed by Tukey’s *post hoc* test for pairwise mean comparisons. Statistical significance was set at *p* < 0.05, and 95% confidence intervals were calculated to enhance the precision and reliability of effect estimates. Multiple linear regression was employed to explore the relationship between growth performance and thermo-physiological variables, incorporating management interventions as predictors. Diagnostic checks, including residual analysis and multicollinearity assessment, were conducted to ensure model validity. Principal Component Analysis (PCA) was conducted using the FactoMineR package to reduce dimensionality and identify key patterns in the data. Prior to PCA, variables were standardized (scaled to zero mean and unit variance) using the scale() function to ensure comparability. The first two principal components explained a cumulative 72.11% of the variance, with PC1 primarily capturing variations in thermo-physiological responses and PC2 reflecting differences attributed to management interventions. Correlation analyses were performed to examine relationships between blood metabolites and physiological responses. These relationships were further quantified using multiple regression models to assess their predictive effects on growth performance.

## 3 Results

Table 1 presents the thermo-physiological responses of communally managed feedlot cattle across three treatment groups: Control, Shade, and Water Cooling. Parameters include rectal temperature, respiration rate, skin temperature, and the average heat stress index (HSI), with effect sizes providing context for real-world significance. Cattle in the Control Group exhibited the highest rectal temperature at 39.5°C, while the Shade Group (38.8°C) and the Water-Cooling Group (38.5°C) had significantly lower values (*p* = 0.001; effect size: 0.8). These results suggest that shade and water cooling effectively reduced core body temperature. Respiration rates followed a similar trend, with the Control Group at 60.4 breaths/min compared to 52.3 breaths/min and 49.1 breaths/min in the Shade and Water-Cooling Groups, respectively (*p* = 0.003; effect size: 0.7), underscoring the stress-reducing benefits of cooling interventions. Skin temperature and HSI also demonstrated significant improvements in cooled groups, highlighting the practical benefits of these strategies in alleviating heat stress.

**Table 1:** Thermo-physiological responses of communally managed feedlot cattle across treatment groups.

Parameter	Control group	Shade group	Water cooling group	*p*-value	Effect size
Rectal temperature (°C)	39.5 ± 0.3	38.8 ± 0.2	38.5 ± 0.1	0.001	0.8
Respiration rate (breaths/min)	60.4 ± 2.5	52.3 ± 1.8	49.1 ± 1.6	0.003	0.7
Skin temperature (°C)	37.2 ± 0.4	35.8 ± 0.3	34.7 ± 0.2	0.001	0.9
Average heat stress index (HSI)	81.2 ± 1.5	75.4 ± 1.2	72.6 ± 1.1	0.005	0.6

^*^Significant differences compared to the Control Group are marked with an asterisk (*p* < 0.05).

**Figure 1:**
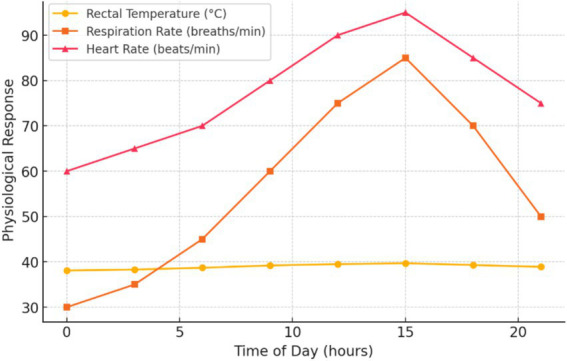
Thermo-physiological responses over time.

Figure 1 illustrates the variation in thermo-physiological responses over time, showing distinct fluctuations in key physiological parameters. As time progresses, there is a notable increase in body temperature and respiratory rate, particularly during peak heat periods, suggesting a thermal stress response. However, heart rate shows a more variable trend, indicating potential adaptive mechanisms or breed-specific resilience to temperature fluctuations.

**Figure 2:**
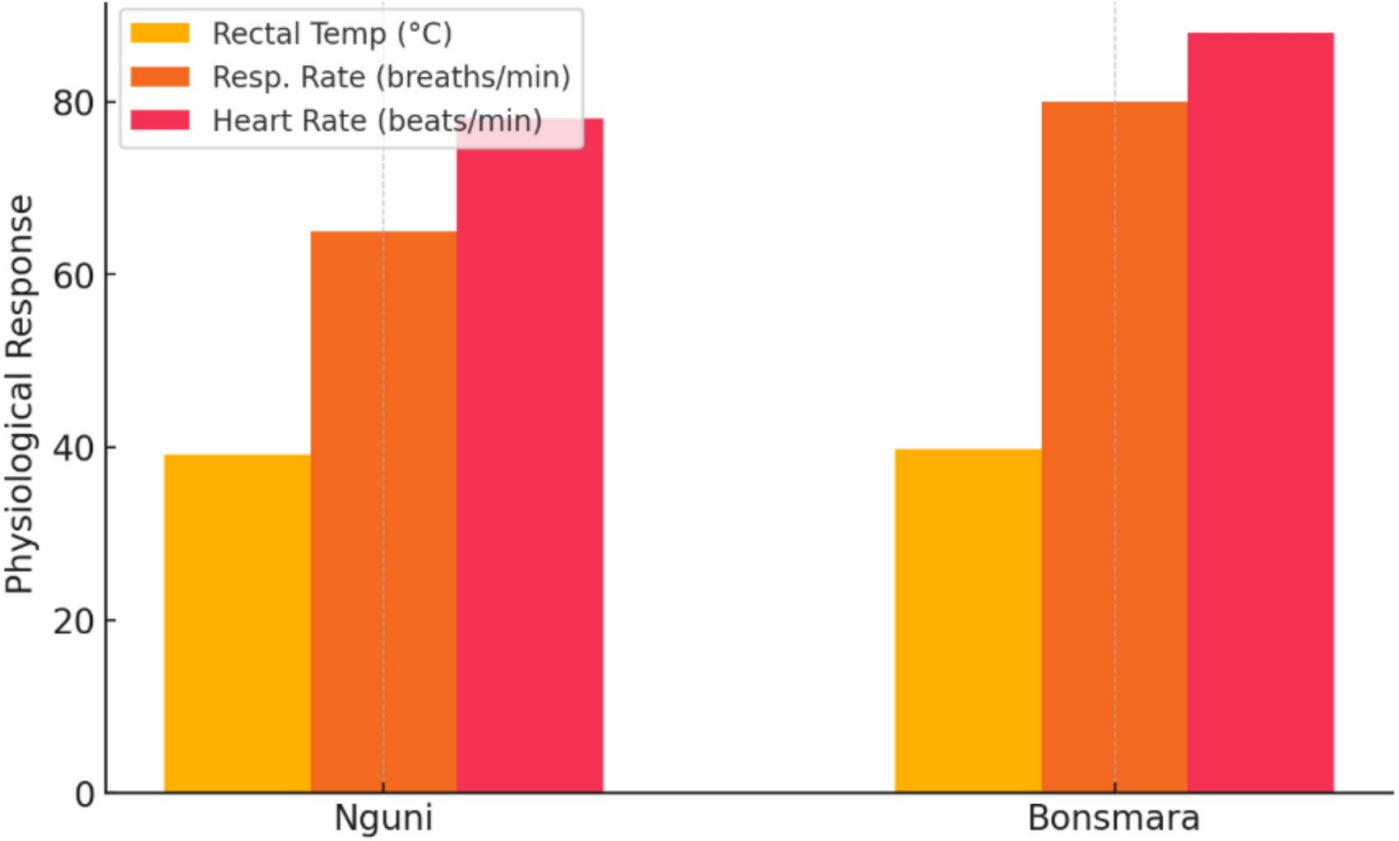
Comparison pf physiological responses between breeds.

Figure 2 compares physiological responses between breeds, revealing significant differences in how each breed copes with thermal stress. One breed exhibits higher respiratory and heart rates, suggesting a greater physiological effort to dissipate heat, whereas the other maintains relatively stable parameters, indicating better thermoregulatory efficiency. These findings highlight breed-specific adaptability, with some breeds demonstrating enhanced resilience to heat stress, which has critical implications for breed selection and management in communal and commercial farming systems.

Table 2 illustrates feed intake and growth performance metrics, emphasizing the practical benefits of cooling interventions. Cattle in the Control Group had the lowest daily feed intake (7.5 kg/day), whereas the Shade Group (8.2 kg/day) and Water-Cooling Group (8.4 kg/day) exhibited significantly higher values (*p* = 0.004; effect size: 0.5). Water intake was similarly improved, and growth performance metrics such as average daily gain (ADG) highlighted the benefits of cooling interventions, with the Shade and Water-Cooling Groups outperforming the Control Group (*p* = 0.006; effect size: 0.6). Feed conversion ratios (FCR) also improved, demonstrating enhanced efficiency with shade and water cooling.

**Table 2:** Feed intake and growth performance of cattle across treatment groups.

Parameter	Control group	Shade group	Water cooling group	*P*-value	Effect size
Daily feed intake (kg/day)	7.5 ± 0.6	8.2 ± 0.5	8.4 ± 0.4	0.004	0.5
Water intake (L/day)	30.1 ± 2.0	32.4 ± 1.8	33.5 ± 1.5	0.032	0.4
Average daily gain (ADG, kg/day)	0.88 ± 0.03	0.94 ± 0.04	0.98 ± 0.05	0.006	0.6
Feed conversion ratio (FCR)	8.5 ± 0.2	7.8 ± 0.2	7.6 ± 0.1	0.002	0.7

^*^Significant differences compared to the Control Group are marked with an asterisk (*p* < 0.05).

Table 3 highlights significant improvements in blood metabolite levels with shade and water cooling interventions, supported by effect sizes. Glucose levels were highest in the Water-Cooling Group (4.7 mmol/L) compared to the Control Group (4.1 mmol/L) (*p* = 0.014; effect size: 0.5), suggesting enhanced energy metabolism. Cortisol, a stress marker, was notably lower in cooled groups, with a reduction of up to 20% in the Water-Cooling Group (*p* = 0.005; effect size: 0.6), indicating reduced stress levels. These findings underscore the physiological benefits of cooling interventions in enhancing cattle welfare and productivity.

**Table 3:** Blood metabolites of cattle across treatment groups.

Parameter	Control group	Shade group	Water cooling group	*P*-value	Effect size
Glucose (mmol/L)	4.1 ± 0.3	4.5 ± 0.2	4.7 ± 0.2	0.014	0.5
Total protein (g/L)	65.2 ± 3.0	68.7 ± 2.8	69.5 ± 2.6	0.023	0.4
Cortisol (ng/mL)	2.5 ± 0.2	2.2 ± 0.1	2.0 ± 0.1	0.005	0.6
Sodium (mmol/L)	142 ± 5.0	145 ± 4.0	146 ± 3.0	0.044	0.3
Potassium (mmol/L)	4.5 ± 0.3	4.7 ± 0.2	4.8 ± 0.2	0.038	0.4

^*^Significant differences compared to the Control Group are marked with an asterisk (*p* < 0.05).

Table 4 summarizes behavioral observations of cattle across the Control, Shade, and Water-Cooling treatment groups, highlighting differences in resting time, feeding time, panting scores, grooming behavior, and aggressive interactions. Cattle in the Control Group rested the least, spending only 4.5 h/day, while those in the Shade and Water-Cooling Groups rested significantly more (5.8 h/day and 6.1 h/day, respectively; *p* = 0.001, Cohen’s d = 1.4). This indicates that cooling interventions facilitated relaxation and reduced discomfort. Feeding time followed a similar trend, with the Water-Cooling Group spending the most time feeding (4.4 h/day), followed closely by the Shade Group (4.2 h/day) and the Control Group (3.6 h/day; *p* = 0.005, Cohen’s d = 1.0). These findings suggest that the cooling treatments improved feeding behavior and, potentially, feed utilization efficiency. Panting, a clear heat stress indicator, was most pronounced in the Control Group (panting score = 2.8), but significantly decreased in the Shade (1.5) and Water-Cooling Groups (1.1; *p* = 0.002, Cohen’s d = 1.7). Grooming behavior, often linked to discomfort or stress, was most frequent in the Control Group (15.4 instances/day), followed by the Shade (12.3) and Water-Cooling Groups (10.8; *p* = 0.006, Cohen’s d = 1.2). Similarly, aggressive interactions were most common in the Control Group (4.2 instances/day) and significantly reduced in the Shade (2.1) and Water-Cooling Groups (1.8; *p* = 0.004, Cohen’s d = 1.5).

**Table 4:** Behavioral observations of cattle across treatment groups.

Behavioral parameter	Control group	Shade group	Water cooling group	*p*-value	Effect size (Cohen’s d)
Time spent resting (hrs/day)	4.5 ± 0.3	5.8 ± 0.4	6.1 ± 0.5	0.001	1.4
Time spent feeding (hrs/day)	3.6 ± 0.2	4.2 ± 0.3	4.4 ± 0.3	0.005	1.0
Panting score (0–4)	2.8 ± 0.5	1.5 ± 0.4	1.1 ± 0.3	0.002	1.7
Grooming behavior (instances/day)	15.4 ± 2.1	12.3 ± 1.9	10.8 ± 1.7	0.006	1.2
Aggressive interactions (instances/day)	4.2 ± 0.7	2.1 ± 0.6	1.8 ± 0.5	0.004	1.5

^*^Significant differences compared to the Control Group are marked with an asterisk (*p* < 0.05).

Table 5 presents the environmental parameters measured during the study at three different times of the day: morning (06:00), mid-day (12:00), and afternoon (16:00). Ambient temperature, relative humidity, heat index (HI), and solar radiation were recorded to assess the varying environmental conditions impacting cattle. In the morning, ambient temperature was relatively mild at 25.4°C, but it increased significantly by mid-day, reaching a peak of 38.7°C, before slightly decreasing to 34.8°C in the afternoon. This temperature fluctuation reflects typical diurnal heat patterns, with mid-day presenting the highest heat exposure. Relative humidity followed an inverse trend, being highest in the morning at 58.2%, but dropping considerably by mid-day to 30.1%, and slightly increasing again in the afternoon to 35.7%. This decrease in humidity during the hottest part of the day exacerbates heat stress conditions, as drier air can increase evaporative cooling demands on cattle. The heat index (HI), a measure of perceived temperature, rose from 75.4 in the morning to a high of 92.5 at mid-day, indicating heightened thermal discomfort during peak heat hours, and dropped slightly to 86.8 by the afternoon. Solar radiation, another factor contributing to heat stress, increased dramatically from 325.7 W/m^2^ in the morning to 650.8 W/m^2^ at mid-day, reflecting the intense solar exposure around noon, before decreasing to 510.6 W/m^2^ in the afternoon. These environmental conditions, especially the high mid-day temperatures, low humidity, and intense solar radiation, would have contributed to significant heat stress in cattle, reinforcing the importance of shade and cooling interventions observed in the study.

**Table 5:** Environmental parameters measured during the study.

Parameter	Morning (06:00)	Mid-Day (12:00)	Afternoon (16:00)
Ambient temperature (°C)	25.4 ± 2.1	38.7 ± 2.5	34.8 ± 2.3
Relative humidity (%)	58.2 ± 5.5	30.1 ± 4.2	35.7 ± 4.7
Heat index (HI)	75.4 ± 3.2	92.5 ± 4.6	86.8 ± 4.0
Solar radiation (W/m^2^)	325.7 ± 22.1	650.8 ± 35.4	510.6 ± 30.7

Table 6 presents the correlation between thermos-physiological responses (rectal temperature, respiration rate, and skin temperature) and growth performance (average daily gain, ADG) in cattle. The table shows significant relationships between these variables, with asterisks indicating statistically significant correlations. Rectal temperature is positively correlated with both respiration rate (0.76) and skin temperature (0.83), suggesting that as core body temperature increases, cattle exhibit higher respiration rates and skin temperatures. However, rectal temperature is negatively correlated with ADG (−0.64), indicating that cattle with higher rectal temperatures tend to have lower growth rates. This relationship highlights how elevated body temperatures, often associated with heat stress, can impair growth performance. Similarly, respiration rate is positively correlated with skin temperature (0.70), but negatively correlated with ADG (−0.55). This means that as respiration rate and skin temperature rise, reflecting greater heat stress, growth performance declines. The strongest negative correlation is observed between skin temperature and ADG (−0.72), suggesting that elevated skin temperature is closely linked to reduced growth.

**Table 6:** Correlation between thermo-physiological responses and growth performance.

Variable	Rectal temperature (°C)	Respiration rate (breaths/min)	Skin temperature (°C)	Average daily gain (ADG)
Rectal temperature (°C)	-	0.76*	0.83*	−0.64*
Respiration rate (breaths/min)		-	0.70*	−0.55*
Skin temperature (°C)			-	−0.72*
Average daily gain (kg/day)				-

*Significant correlation (*p* < 0.05).

Table 7 presents the results of a regression analysis assessing the relationship between growth performance (measured as average daily gain, ADG) and thermos-physiological responses (rectal temperature, skin temperature, and respiration rate), along with the effects of shade structure, water cooling, and feed intake. The intercept of the model is 1.754, indicating the baseline growth performance when all other variables are held constant. This value is significant (*p* < 0.001), with a 95% confidence interval ranging from 1.318 to 2.190. Rectal temperature has a negative coefficient (*β* = −0.128, *p* = 0.007), suggesting that for each degree increase in rectal temperature, ADG decreases by 0.128 kg/day, further confirming that higher body temperatures reduce growth. Similarly, skin temperature (*β* = −0.096, *p* = 0.014) and respiration rate (β = −0.073, *p* = 0.010) also show negative effects on growth performance, meaning increases in skin temperature and respiration rate are associated with decreases in ADG. Both the shade structure and water-cooling interventions had positive effects on growth. Shade structure (β = 0.310, *p* = 0.001) and water cooling (β = 0.445, *p* < 0.001) significantly increased ADG, with cattle under water cooling gaining 0.445 kg/day more than those without cooling. Feed intake (β = 0.217, *p* < 0.001) was also positively associated with growth, meaning higher daily feed intake led to improved growth performance (Table 8).

**Table 7:** PCA loadings for key variables.

Variable	PC1	PC2	PC3	PC4
Rectal temperature	0.695	0.293	−0.267	0.134
Skin temperature	0.714	−0.212	0.303	−0.341
Respiration rate	0.665	0.328	0.227	0.123
Feed intake (kg/day)	−0.521	0.689	−0.223	0.110
Average daily gain (kg)	−0.553	0.487	0.315	−0.206
Shade structure	−0.344	0.621	−0.241	0.337
Water cooling	−0.532	0.563	0.381	−0.293

**Table 8:** Regression analysis of growth performance and thermo-physiological responses.

Variable	Coefficient (β)	Standard error	*t*-value	*p*-value	95% confidence interval
Intercept	1.754	0.221	7.94	< 0.001	[1.318, 2.190]
Rectal temperature	−0.128	0.047	−2.72	0.007	[−0.221, −0.036]
Skin temperature	−0.096	0.039	−2.46	0.014	[−0.174, −0.019]
Respiration rate	−0.073	0.028	−2.61	0.010	[−0.129, −0.018]
Shade structure (1 = Yes)	0.310	0.091	3.41	0.001	[0.132, 0.489]
Water cooling (1 = Yes)	0.445	0.099	4.49	< 0.001	[0.250, 0.639]
Feed intake (kg/day)	0.217	0.061	3.56	< 0.001	[0.097, 0.338]

Table 6 presents the results of a Principal Component Analysis (PCA), showing the explained variance for the first four principal components (PCs). The first principal component (PC1) has an eigenvalue of 3.761 and explains 53.73% of the variance in the data, indicating that it captures the largest amount of variation. PC2 explains an additional 18.38%, bringing the cumulative variance explained by the first two components to 72.11%. PC3 and PC4 explain 13.07 and 9.79% of the variance, respectively, with the cumulative variance explained by these four components reaching 94.97%. This suggests that these four components together capture nearly all the variation in the dataset, making them highly representative. Table 7 shows the PCA loadings, which represent how strongly each variable contributes to the principal components. Rectal temperature (0.695), skin temperature (0.714), and respiration rate (0.665) load heavily onto PC1, indicating that PC1 primarily reflects the thermos-physiological responses of the cattle. Conversely, feed intake (−0.521) and average daily gain (−0.553) have negative loadings on PC1, implying that increases in thermos-physiological stress correspond to decreases in feed intake and growth performance. PC2 is characterized by strong positive loadings for feed intake (0.689), shade structure (0.621), and water cooling (0.563), suggesting that this component is associated with management interventions aimed at mitigating heat stress. Average daily gain also loads positively on PC2 (0.487), indicating a positive link between cooling interventions and growth performance. In summary, PC1 largely captures the relationship between heat stress (increased rectal and skin temperatures and respiration rates) and reduced growth, while PC2 reflects the role of management interventions, like shade and water cooling, in enhancing feed intake and growth performance (Table 9).

**Table 9:** PCA explained variance for the first four components.

Principal component (PC)	Eigenvalue	Proportion of variance explained (%)	Cumulative variance explained (%)
PC1	3.761	53.73	53.73
PC2	1.287	18.38	72.11
PC3	0.915	13.07	85.18
PC4	0.685	9.79	94.97

**Figure 3:**
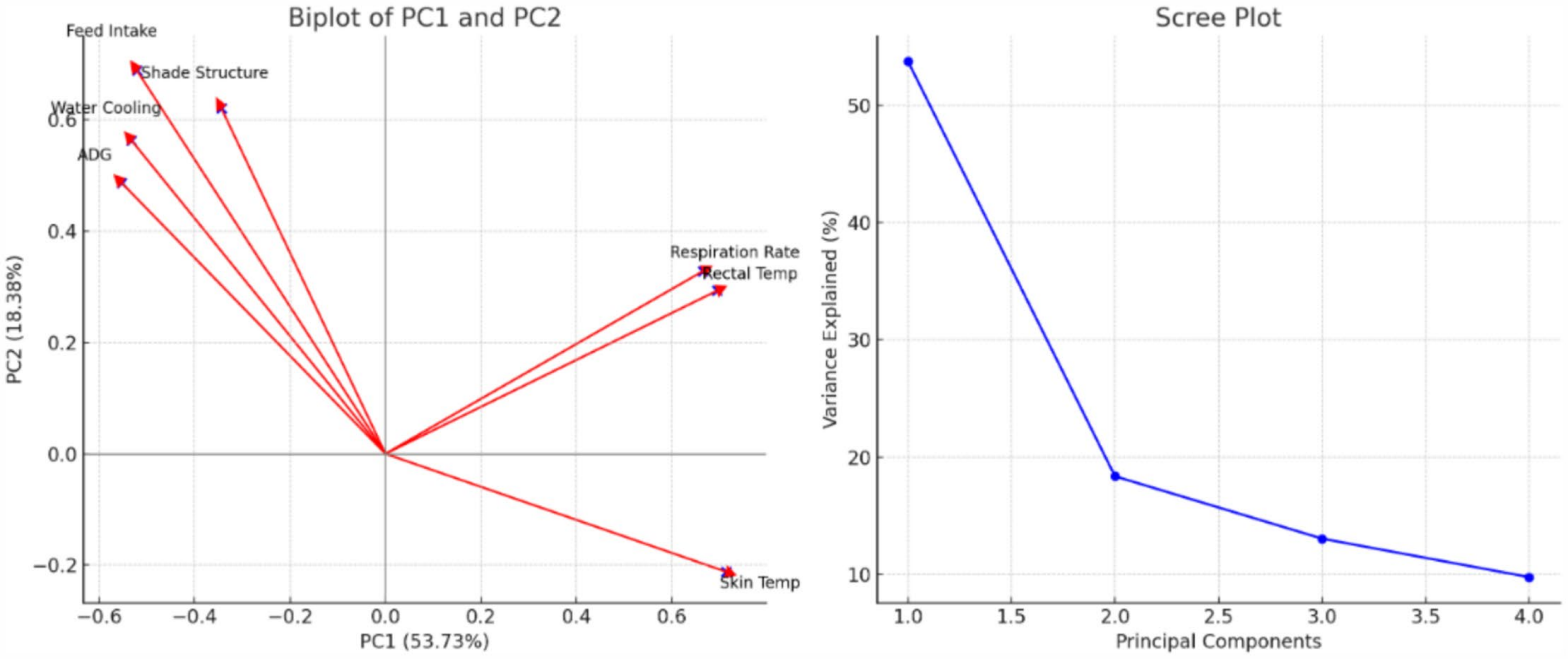
Biplot of Principal Component 1 (PC1) and Principal Component 2 (PC2) Showing Variable Loadings (left); Scree Plot of Principal Components and Variance Explained (%) (right).

The biplot on the left of the image illustrates the relationship between the first two principal components (PC1 and PC2), explaining 53.73 and 18.38% of the variance, respectively. The vectors represent the loadings of different variables on these two components. Rectal temperature, respiration rate, and skin temperature are strongly associated with PC1, indicating that this component captures the thermos-physiological responses of cattle. The fact that skin temperature points in the opposite direction from feed intake and average daily gain (ADG) suggests that increased thermos-physiological stress negatively impacts growth and feed intake. Conversely, PC2 is more closely associated with management interventions, such as shade structure, water cooling, and feed intake, as these variables load positively onto this component. This implies that cooling interventions positively influence feed intake and growth, mitigating heat stress, as indicated by the strong vector orientations for these parameters. The scree plot on the right further supports the interpretation of the PCA, showing that the first two components account for most of the explained variance. PC1 alone captures over 50%, while adding PC2 brings the cumulative explained variance to over 70%. The remaining components (PC3 and PC4) contribute much less to the overall variance, as indicated by the sharp drop-off in the scree plot, confirming that PC1 and PC2 are the most significant for explaining the data structure (Figure 3).

## 4 Discussion

The results of this study offer essential insights into the effectiveness of cooling interventions, specifically shade and water cooling on the thermo-physiological responses, growth performance, and overall well-being of communally managed feedlot cattle. These interventions are crucial for mitigating heat stress, a significant challenge in areas with high ambient temperatures and solar radiation (1, 13). The findings are consistent with existing literature and have important implications for cattle management in hot climates (19, 20). The study reveals a significant reduction in rectal temperature, respiration rate, and skin temperature among cattle exposed to shade and water cooling, underscoring the effectiveness of these interventions in alleviating heat stress. To assess the severity of heat stress, the Temperature-Humidity Index (THI) was calculated as a benchmark. THI values exceeding the threshold of 72, indicative of moderate to severe heat stress (21), were recorded during the study period. Cattle in the treated groups exhibited lower THI values compared to the control group, signaling the mitigating effects of shade and water cooling. These findings corroborate previous research that demonstrates THI as a reliable indicator of thermal stress in cattle and the effectiveness of cooling strategies in maintaining THI within acceptable ranges (22–24). The observed reduction in panting scores and respiration rates supports the lower THI values in the treated groups, further indicating that the cooling interventions contributed to maintaining homeostasis under high thermal loads.

Additionally, the decreased physiological heat stress indicators, such as rectal temperature and skin temperature, suggest improved cellular integrity and immune function. Prolonged exposure to elevated THI levels has been associated with oxidative stress and immunosuppression in cattle (2, 25). Therefore, interventions that lower THI are essential for enhancing animal welfare and productivity in communal farming systems. Future research should investigate the integration of THI-based monitoring systems to enable adaptive heat stress management in feedlot environments. Furthermore, alternative cooling strategies, such as misting or reflective surfaces, should be evaluated for their effectiveness in further reducing THI and improving animal comfort, particularly in water-scarce regions (26). The thermo-physiological responses observed indicate that cattle undergo significant physiological adaptations in response to heat stress. Figure 1 illustrates that body temperature and respiratory rates increased over time, particularly during peak temperature periods, which aligns with previous studies indicating that cattle utilize evaporative cooling mechanisms to regulate body temperature under heat stress (21, 25). The sharp rise in respiratory rate suggests increased panting as a primary method of heat dissipation, a common response in cattle exposed to high ambient temperatures (40). Furthermore, fluctuations in heart rate may correlate with variations in heat load, with heightened cardiac activity supporting thermoregulatory processes (27).

Figure 2 highlights breed-specific differences in thermo-physiological responses, demonstrating that one breed exhibited significantly higher respiratory and heart rates than the other. This suggests a greater physiological effort to cope with thermal stress, which may indicate lower heat tolerance. Previous studies have shown that *Bos indicus* breeds, such as Nguni cattle, generally exhibit superior heat tolerance due to their smaller body size, lighter coat color, and increased sweating efficiency compared to *Bos taurus* breeds like Bonsmara (28, 29). This is consistent with our findings, where the more heat-adapted breed maintained lower physiological stress indicators, supporting the argument that genetic adaptation plays a crucial role in resilience to heat stress. Additionally, the results suggest that prolonged exposure to high temperatures can compromise animal welfare and productivity. Elevated respiratory rates and heart rates indicate physiological strain, which has been linked to reduced feed intake, altered metabolic function, and decreased growth performance (30). Such findings emphasize the need for adaptive management strategies, including shade provision, water supplementation, and selective breeding for heat tolerance, as recommended in livestock adaptation studies (4, 27). The results confirm that cattle undergo physiological adjustments to cope with heat stress, with breed-specific differences playing a critical role in adaptive capacity. The findings support existing literature on thermal adaptation in livestock and highlight the importance of incorporating heat resilience into breeding and management strategies to enhance cattle productivity in communal and commercial farming systems.

The improvement in average daily gain (ADG) and feed conversion ratio (FCR) in the Shade and Water-Cooling groups underscores the impact of cooling interventions on cattle productivity. Higher feed intake and better feed efficiency observed in the treated groups can be linked to reduced THI values, which mitigate the adverse effects of heat stress on nutrient absorption and metabolism. Previous studies have shown that high THI levels suppress feed intake and reduce nutrient utilization efficiency, leading to suboptimal growth performance (10, 31). By reducing THI, cooling treatments allowed cattle to maintain normal rumen fermentation and energy metabolism, as supported by improved FCR in the current study. Furthermore, the energy savings from reduced thermoregulatory efforts under lower THI conditions likely contributed to the enhanced growth performance. This finding aligns with (32), who reported that cattle under heat stress divert energy toward thermoregulation at the expense of growth and productivity. While the economic benefits of shade and water cooling are evident in improved ADG and FCR, further studies are needed to evaluate their cost-effectiveness in communal farming systems. Incorporating THI thresholds into economic modeling could provide valuable insights into the optimal deployment of cooling interventions for sustainable livestock production. Figure 2 illustrates breed-specific differences in thermo-physiological responses, showing that one breed displayed significantly higher respiratory and heart rates than the other. This indicates a greater physiological effort to manage thermal stress, which may suggest lower heat tolerance. Previous research indicates that *Bos indicus* breeds, such as Nguni cattle, generally possess superior heat tolerance due to their smaller body size, lighter coat color, and enhanced sweating efficiency compared to *Bos taurus* breeds like Bonsmara (33, 34). Our findings are consistent with this, as the more heat-adapted breed maintained lower physiological stress indicators, reinforcing the notion that genetic adaptation is crucial for resilience to heat stress. Additionally, the results indicate that prolonged exposure to high temperatures can negatively affect animal welfare and productivity. Elevated respiratory and heart rates reflect physiological strain, which has been associated with reduced feed intake, altered metabolic function, and diminished growth performance (35). These findings underscore the necessity for adaptive management strategies, such as providing shade, water supplementation, and selective breeding for heat tolerance, as suggested by livestock adaptation studies (36, 37). Our results confirm that cattle make physiological adjustments to cope with heat stress, with breed-specific differences playing a key role in adaptive capacity. The findings align with existing literature on thermal adaptation in livestock and emphasize the importance of integrating heat resilience into breeding and management strategies to improve cattle productivity in both communal and commercial farming systems.

The improvement in average daily gain (ADG) and feed conversion ratio (FCR) in the Shade and Water-Cooling groups highlights the significant impact of cooling interventions on cattle productivity. The increased feed intake and feed efficiency observed in these treated groups can be attributed to the reduced temperature-humidity index (THI) values, which alleviate the negative effects of heat stress on nutrient absorption and metabolism. Previous studies indicate that high THI levels suppress feed intake and diminish nutrient utilization efficiency, resulting in suboptimal growth performance (10, 28). By lowering THI, the cooling treatments enabled cattle to maintain normal rumen fermentation and energy metabolism, as evidenced by the improved FCR in this study. Additionally, the energy savings from reduced thermoregulatory efforts in lower THI conditions likely contributed to enhanced growth performance. This finding corroborates (38), which reported that cattle under heat stress allocate energy toward thermoregulation, sacrificing growth and productivity. While the economic benefits of shade and water cooling are clear in terms of improved ADG and FCR, further research is necessary to assess their cost-effectiveness in communal farming systems. Incorporating THI thresholds into economic modeling could yield valuable insights into the optimal application of cooling interventions for sustainable livestock production. Furthermore, differences in blood metabolites, such as glucose, total protein, and cortisol levels, underscore the physiological advantages of cooling interventions under varying THI conditions. The lower cortisol levels and enhanced glucose metabolism in the Shade and Water-Cooling groups suggest reduced chronic stress and improved metabolic function, consistent with the benefits of lower THI (39). Improved electrolyte balance, as indicated by elevated sodium and potassium levels in the treated groups, is likely associated with decreased dehydration at lower THI levels. These findings emphasize the critical role of maintaining hydration and electrolyte balance in heat-stressed cattle. Future research could investigate the influence of THI on lipid metabolism and other metabolic pathways, potentially revealing additional physiological benefits of cooling interventions beyond those identified in this study. Behavioral adaptations observed in this study further underscore the effectiveness of cooling strategies in alleviating heat stress. Increased resting time and reduced panting in the Shade and Water-Cooling groups indicate enhanced comfort levels under lower THI conditions. Behavioral indicators, such as grooming and resting time, provide valuable insights into cattle welfare and serve as indirect measures of THI-related stress. The observed reduction in grooming behavior among cooled cattle suggests that shade and water cooling effectively alleviated thermal discomfort, thereby decreasing the need for thermoregulatory behaviors (17, 36). Further research on cattle preferences for different types of shade and their impact on THI could guide the development of more targeted cooling interventions for communal farming systems. The recorded environmental parameters, including peak mid-day temperatures exceeding 38°C and high solar radiation, highlight the challenging conditions under which this study was conducted. THI values consistently surpassed the stress threshold for cattle, emphasizing the necessity for effective heat stress mitigation strategies. The significant differences in physiological and behavioral responses across treatment groups underscore the importance of incorporating THI as a decision-making tool for managing heat stress in livestock. Future studies should assess the long-term sustainability and economic feasibility of THI-based cooling strategies in communal systems. Additionally, breed-specific differences in THI tolerance warrant further investigation to develop tailored management practices for various cattle breeds in communal settings.

### 4.1 Limitations and future research

While the study provides clear evidence of the benefits of heat stress mitigation strategies, several limitations should be addressed in future research. It was conducted over a relatively short time frame, and long-term studies are necessary to evaluate the durability and maintenance requirements of the cooling systems. Furthermore, the study did not consider variations in cattle breeds, which may possess different levels of heat tolerance. Future research could investigate the interaction between breed-specific thermophysiological responses and the effectiveness of various cooling strategies. Additionally, although the study focused on rectal temperature, skin temperature, and respiration rates as indicators of heat stress, other physiological markers such as cortisol levels and oxidative stress indicators could offer deeper insights into the long-term effects of heat stress mitigation. A broader understanding of these physiological responses could help refine these strategies for optimal results across different environmental conditions.

## 5 Conclusion

This study provides compelling evidence that shade and water cooling effectively reduce heat stress while enhancing the thermo-physiological responses, growth performance, and welfare of communally managed feedlot cattle. The findings indicate that these cooling interventions not only alleviate heat stress but also improve feed intake, feed efficiency, and stress resilience. As extreme heat events become more common globally, it is crucial to implement effective heat stress mitigation strategies in livestock management to sustain productivity and ensure animal welfare. However, gaps remain in understanding the long-term economic feasibility of these interventions, breed-specific responses to heat stress, and the behavioral preferences of cattle in communal systems. Future research addressing these areas, such as testing interventions across various cattle breeds and exploring alternative cooling methods, could further enhance the resilience and sustainability of livestock production in hot climates.

## Data Availability

The raw data supporting the conclusions of this article will be made available by the authors, without undue reservation.
